# Systematic review of factors influencing oral health-related quality of life in children in Africa

**DOI:** 10.4102/phcfm.v11i1.1943

**Published:** 2019-07-24

**Authors:** Yolanda Malele-Kolisa, Veerasamy Yengopal, Jude Igumbor, Cathrine B. Nqcobo, Tshakane R.D. Ralephenya

**Affiliations:** 1 Department of Community Dentistry, School of Oral Health Sciences, University of the Witwatersrand, Johannesburg, South Africa; 2 School of Public Health, University of the Witwatersrand, Johannesburg, South Africa; 3 Department of Paediatric and Restorative Dentistry, School of Oral Health Sciences, University of the Witwatersrand, Johannesburg, South Africa

**Keywords:** oral health, oral health-related quality of life, factors, children, Africa

## Abstract

**Background:**

Oral health-related quality of life (OHRQoL) is influenced by cultural and societal context. Existing OHRQoL children measurement tools have been conceptualised in high-income countries. Probing whether the factors influencing OHRQoL are context-reliant in the African setting is necessary and is the purpose of the current review.

**Aim:**

To investigate if the factors influencing OHRQoL are context-reliant.

**Methods:**

Seven databases were searched using search terms (‘oral health’; and ‘quality of life’, ‘health-related quality of life’, ‘patient-reported outcomes’, ‘well-being’; and ‘child*’, ‘adolescents’, ‘teen*’, ‘youth’; and ‘determinants’, ‘factors’, ‘predictors’; and ‘oral health quality of life tools/instruments/scales’; and ‘Africa*’). Abstracts identified were exported to a reference software manager. Three of the authors used specific selection criteria to review, firstly, 307 abstracts and, secondly, 30 full papers. Data were extracted from these papers using a pre-designed data extraction form, after which quantitative synthesis of data was performed.

**Results:**

Key factors influencing OHRQoL followed an existing conceptual framework where environmental and individual factors in the form of socio-economic status (SES), area of residence and children psyche status, and the presence of any oral condition other than dental caries were reported among child populations in Africa.

**Conclusion:**

There is preliminary evidence to suggest an association between individual factors such as children’s psyche and oral problems, excluding dental caries, and environmental determinants such as area of residence and SES in children’s OHRQoL in African children. The finding that dental caries was not a key factor in child-oral health is unexpected. There seemed to be a contextual viewpoint underpinning the current OHRQoL frameworks and OHRQoL was context-reliant.

## Introduction

The FDI World Dental Federation vision 2020 defines oral health as:

multi-faceted and includes the ability to speak, smile, smell, taste, touch, chew, swallow and convey a range of emotions through facial expressions with confidence and without pain, discomfort and disease of the craniofacial complex.^1^

The definition connects with Locker and Allen’s concept of oral health-related quality of life (OHRQoL), which is explained simply and loosely as ‘impact of oral conditions on daily functioning and well-being’.^2^ Oral health-related quality of life deals with effects and symptoms that vary in intensity and importance. ‘Some maybe life threatening (e.g. oral cancers), some chronic (caries, periodontitis), some aesthetic (malocclusion, fluorosis), while some are acute and painful (toothache, pulpitis, oral mucosal lesions, extractions)’, according to Hernández et al.^3^ Conceptualisation of OHRQoL is context-reliant as culture and society shapes an individual’s belief system and influences how health and illness is viewed.^4^ Studies conducted on children’s oral conditions have generally reported a poorer OHRQoL because of the oral conditions.^5,6,7,8^ Herdman et al. purport that OHRQoL measurement domains important to one culture may not be equally relevant in all cultures.^9^

Recently, research on children’s oral conditions and OHRQoL globally has described the factors influencing OHRQoL.^10,11,12,13^ Oral health-related quality of life research in children is fairly recent in Africa.^14,15,16,17^ Existing children tools that measure OHRQoL have been developed in a non-African setting; however, some have been adapted and tested in the African setting. According to Traebert et al., different existing tools showed discrepancies when applied to different cultural backgrounds when probing the concept of ethnicity and socialisation despite translation.^18^ With the amount of OHRQoL literature available on children in the African setting, no consolidating integrative review has been conducted regarding the factors influencing OHRQoL. It is, thus, necessary to probe whether the factors influencing OHRQoL are context-reliant.

Consideration of sociocultural contexts and factors affecting children’s OHRQoL is important as OHRQoL is a social construct. The majority of health-related quality of life (HRQoL) models are based on the biomedical and psychological dimensions of health such as those in the International Classification of Impairments, Disabilities and Handicaps model by the World Health Organization (ICIDH, 1980). The Locker model is a type of such HRQoL model that is based on the WHO-ICIDH.^19^ The Locker model is the dental adaptation of the WHO-ICIDH, and the model hypothesises that oral disease will result in ‘pain’, ‘impairment’ and functional limitation. These constructs will, in turn, lead to physical and psychological disability, handicap, and thus affect the overall OHRQoL.^19^ The Locker model places its emphasis on multidimensionality of health positioned around the biomedical model and incorporates a psychosocial dimension.

**FIGURE 1:**
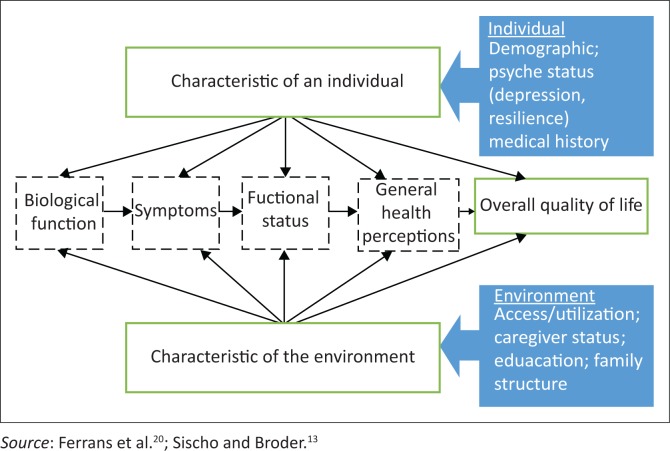
A conceptual framework of health-related quality of life and its determinants.

In 2005, Ferrans et al. developed an OHRQoL conceptual framework which posits that the biological–symptom–functional status complex is directly influenced by both individual and environmental characteristics and together they influence the general perceptions of health and overall OHRQoL (Figure 1).^20^ Sischo and Broder even unpack the Ferrans et al.’s model further when applying it to oral health by expanding on the individual and environmental characteristics.^13,20^ On the contrary, a newer model, such as the International Classification of Functioning, Disability and Health (ICF), has an elaborate classification which includes holistic non-sick-based dimensions such as functioning and health. These models and theories provide a guiding point of view when interrogating the factors that may influence OHRQoL among African children.

Oral health-related quality of life in children is particularly important as childhood development involves phases that are dominated by life changes notwithstanding the added burden of living with oral diseases.^4,21,22^ Wallander et al. noted that HRQoL has a dual subjective–objective elements underlined by a time in one’s life.^4^ Children generally tend to have higher prevalence of oral conditions.^23^ A review of the life stages before adulthood has not been thoroughly investigated in the broad field of OHRQoL in general and in the African context in particular.

This review will be the first to our knowledge that seeks to explore and integrate available literature on the factors influencing OHRQoL in children in the African setting. The review will provide more insight into understanding the phenomenon and to add a voice to the integrated management of children’s oral conditions and the related impact on HRQoL.

## Methods

This review addresses the following question: What are the determinant factors that influence OHRQoL among children in Africa? It seeks to establish, through the available literature, factors influencing OHRQoL in children in Africa. The objective is to integrate and summarise the factors influencing OHRQoL in children diagnosed with oral conditions or problems throughout Africa. The systematic review was registered with PROSPERO number: CRD42017056759. The inclusion requirements, as shown in Table 1, were followed. No publication date limit was set as this type of review has not been done before.

**TABLE 1:** Inclusion exclusion criteria of articles in the review.

Article characteristics	Included	Excluded
Publication type	Peer-reviewed, full-text, English language articles dated till 2017	Non-English articles Editorials, review articles, letters, practice guidelines, other guideline documents, conference abstracts, conference reports, news articles
Study design	Any study design with the measurement or assessment of OHRQoL using validated tools	-
Study population and study setting	All children’s studies and where mothers were used as a proxy for young children Studies with overlap up to adulthood (e.g. 21 years) were included and the outcome referring to children only was used in the data extraction The study site was anywhere in Africa	Any study with adult participants only The study setting was not Africa
Condition of interest	Factors influencing OHRQoL	Factors pertaining to dental anxiety, satisfaction and any related topic other than OHRQoL
Outcome	OHRQoL using validated measures	Did not use validated measures for OHRQoL

OHRQoL, Oral health-related quality of life.

A systematic literature search of multiple databases with published English language articles in PubMed, CINAHL, EMBASE (Excerpta Medical Database), MEDLINE, EBSCOhost, OVID and PsychINFO was performed with key words: #1 ‘oral health’; and #2 ‘quality of life’, ‘health-related quality of life’, ‘patient-reported outcomes’, ‘well-being’; and #3 ‘child*’, ‘adolescents’, ‘teen*’, ‘youth’; and 4# ‘determinants’, ‘factors’, ‘predictors’; and #5 ‘oral health quality of life tools/instruments/scales’; and #6 ‘Africa*’.

### Data synthesis

A multi-level search process was used starting with the screening which included an independent review of titles by three of the authors of this article (Y.M.-K., C.B.N., T.R.D.R.). This was followed by a review of abstracts and full articles selected after the abstract review. Thereafter a reference list of selected full articles was reviewed to retrieve more articles. Identified studies that met the publication criteria were grouped into one of the following categories: experimental studies, cohort studies, case control studies and cross-sectional observational studies. These studies were then assessed independently for methodological validity by three reviewers, prior to inclusion in the review, using the corresponding checklist developed by the Joanna Briggs Institute tool for cross-sectional, random controlled trials and cohort studies. Each of the tools has the components related to selection criteria, validity and reliability of exposure and outcome variables, confounders, objective measurement of outcome and appropriate statistical tests used. Any disagreements that arose between the reviewers were resolved through discussion among the three reviewers. Where required, a consensus of two out of three was the deciding factor. Following assessment of methodological quality, the papers were grouped according to whether they are quantitative, qualitative designs or opinion-based. A data extraction tool was developed specifically for quantitative research data. None of papers retrieved used qualitative study designs. Three reviewers independently performed data extraction.

Quantitative studies were classified using a table noting publication year, study setting, participants’ age, sample size, study design, OHRQoL outcome and the underpinning predictors or factors or determinants, odds ratio (OR) and corresponding confidence intervals (CIs) (Table 2). If more than one study reported the same factors influencing OHRQoL, the results were pooled into a statistical meta-analysis. Where statistical pooling was not possible, the findings were presented in the form of a narrative analysis. The initial step of meta-analysis was to assess the heterogeneity of the studies to be pooled. When studies were heterogeneous, a random effect model was assumed to account for unequal weighting of the studies. Heterogeneity was quantified using *I*^2^ statistic. A variation of 25% or less meant that there was a low heterogeneity of studies, 26% – 50% meant moderate and 56% – 75% implied high heterogeneity. Thereafter a final outcome of interest was read from ORs. Forest plots were created displaying the results from individual studies, together with the summary and 95% CI estimated in the meta-analyses. Random effect models were assumed because of the following reasons: (1) the studies had a wide age range of child participants, (2) the studies were conducted in different African countries and (3) studies used different validated tools measuring OHRQoL. Meta-analyses were conducted separately for the same factors. For instance, all studies where dental caries, area of residence, oral problems and socio-economic status (SES) were factors were analysed separately.

**TABLE 2:** List of studies retrieved from the database search.

Authors	Publication year	Study setting	Facility	Sample age	Sample size	Study design	OHRQoL outcome (%)	OHRQoL tool (Box 1)
Åstrøm et al.^15^	2016	Tanzania	School	12–15; 16–21 years	2412	Cohort study	50.7%	OIDP
Birungi et al.^28^	2016	Uganda	Community-based	Mother–child pairs (5 years)	863–765	RCT	23.1 and -26.4	ECOHIS
Chukwumah et al.^25^	2016	Nigeria	Local government area schools	12–15 years	1790	C-S study	56.5	C-OIDP
Tagelsir et al.^29^	2013	Sudan	School	6–18 years	79 of 92	C-S study	15.9	C-OIDP
Masumo et al.^32^	2012	Tanzania and Uganda	Mother and child health clinic	Mother–child pairs (6–36 months)	1221	C-S study	32.5–36.5	ECOHIS
Mbawalla et al.^7^	2011	Tanzania	School	12–17 years	2678	C-S study	39.65	C-OIDP
Nurelhuda et al.^30^	2010	Sudan	School	12 years	1109	C-S study	54.6	C-OIDP
Mashoto et al.^31^	2009	Tanzania	School	10–14; 15–19 years	2465	C-S study	36.2	OIDP
Mtaya et al.^8^	2007	Tanzania	School	13 years	1601	C-S study	28.6	C-OIDP
Åstrøm and Okullo^16^	2003	Uganda	-	13–15; 16–19 years	1146	C-S study	62	OIDP
Mashoto et al.^24^†	2010	Tanzania	School	10–14; 15–19 years	1306	RCT	35.6	C-OIDP
Hobdell et al.^26^†	2009	South Africa, UK, US	Schools	15–16; 11–12; 40+ years	525	C-S study	SA: 49.5 US: 52.6 UK: 28.8	OIDP
Wandera et al.^27^†	2009	Uganda	Mother and child health clinic	Mother–child pairs (6–36 months)	816	C-S study	Child : 37.7 Family: 47.1	ECOHIS
Robinson et al. (2005)^60^†	2005	Uganda	School	12 years	174	C-S study	Sum: 39.9	CPQ_11–14_
Åstrøm and Mashoto^14^†	2002	Tanzania	School	12–20 years	492	C-S study	-	OIDP Dissatisfaction (oral condition and dental appearance)

UK, United Kingdom; US, United States

† , Excluded from meta-analysis but included in narrative analysis.

C-S, cross-sectional study; RCT, randomised controlled trial; OHRQoL, oral health-related quality of life; OIDP, Oral Impacts on Daily Performances; C-OIDP, Child-Oral Impacts on Daily Performances; ECOHIS, Early Childhood Oral Health Impact Scale; CPQ_11–14_, Child Perceptions Questionnaire.

### Ethical considerations

Ethical approval was obtained from the Human Research Ethics Committee of the University of the Witwatersrand (Clearance certificate no. M141150).

## Results

**FIGURE 2:**
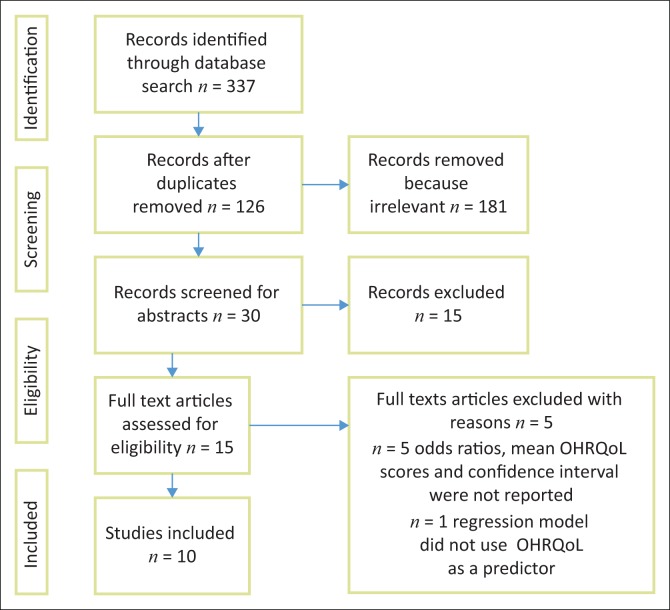
Preferred Reporting Items for Systematic Reviews and Meta-Analyses (PRISMA) flowchart depicting the flow of information through different phases.

A total of 337 articles were retrieved from all databases. Following identification and screening, only 15 articles were assessed for eligibility (Figure 2). Of the 15 eligible articles, four were excluded from the narrative or meta-analysis despite using validated OHRQoL tools because they did not perform regression analysis to report on the factors influencing OHRQoL.^24,25,26,27^ The regression analysis was crucial to ascertain the likelihood of independent variables influencing OHRQoL. One article was also excluded because it reported ORs, but the OHRQoL was not the dependant variable in the regression model but the presence or absence of dental fluorosis.^14^ The study reported dissatisfaction with oral condition (dental fluorosis) and appearance as the outcome in the logistic regression instead of OHRQoL.^14^ Finally, only 10 articles were included for analysis.

### Characteristics of the studies finally included in the review

The main characteristics of the studies in the review are shown in Table 2. All of the 10 studies used validated tools to measure OHRQoL outcomes (Box 1). All studies were quantitative in design; nine were conducted in the East African region (Tanzania, Uganda and Sudan); they were also similar in sharing the same authors or co-authors.^7,8,15,16,25,28,29,30,31^ Only one study was conducted in West Africa (Nigeria).^25^ The study designs were biased towards cross-sectional studies with eight out of 10 (80%) using that design. The measure of association of interest was OR and the related CIs.

BOX 1 Studies that used validated tools measuring oral health-related quality of life.

1. Family Impact Scale (FIS) (Locker et al. 2002)^61^
2. Child Perceptions Questionnaire (CPQ_6–7_); (CPQ_8–10_) and (CPQ_11–14_) (Jokovic et al. 2004a; Jokovic et al. 2004b; Jokovic et al.2002)^62,63,64^
3. Child-Oral Impacts on Daily Performances (Child-OIDP) (Gherunpong et al. 2004)^65^
4. Early Childhood Oral Health Impact Scale (ECOHIS) (Pahel et al. 2007)^66^

Note: With reference to Table 2.

### Factors influencing oral health-related quality of life where studies were fitted into forest plots for meta-analysis

#### Dental caries

**FIGURE 3:**
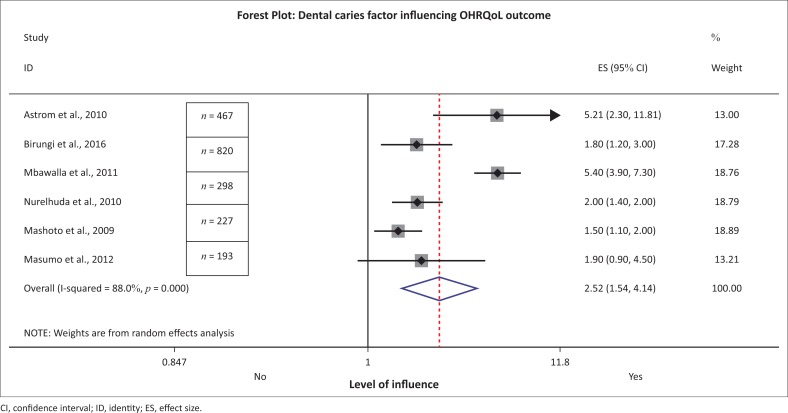
Forest plot for dental caries and oral health-related quality of life.

Six studies in this review reported the presence of dental caries as a factor influencing OHRQoL.^7,15,25,30,31,32^ Four of the studies were performed in Tanzania with a total of 7617 children and they reported the variable dental caries experience measured by DMFT/dmft (Decayed Missing Filled Teeth/decayed missing filled teeth) index. In both primary and secondary dentition, the caries index ranged from being 1.5 times more likely to up to 5.2 times more likely to lead to a poorer OHRQoL among children. Studies based in the Sudan and Uganda reported mid-range ORs of 2.0 (95% CI: 1.4–2.6) and 1.8 (95% CI: 1.2–3.0), respectively (*n* combined = 1874) (Figure 3).

Dental caries, when fitted for meta-analysis, showed an overall high level of influence with regard to OHRQoL in total combined studies of 9491 child participants. However, the results of the *I*^2^ statistics showed that the studies were heterogeneous (88%, *p* = 0.000) (Figure 2). This implies that the studies cannot be pooled because of high heterogeneity. Even though studies all used validated OHRQoL (Table 2), responses are still subjective heterogeneity and the overall conclusion from meta-analysis is explained later in the discussion.

#### Oral conditions other than dental caries

**FIGURE 4:**
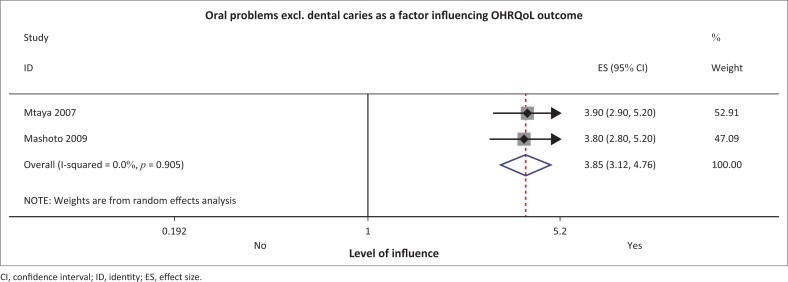
Forest plot for oral problems other than dental caries.

Mashoto et al. in their Tanzanian study reported, with a total of 1745 children with a mean age of 13.8 years (s.d. 1.67), that having any oral condition other than dental caries increased the odds of a poorer OHRQoL (OR: 3.8, 95% CI: 2.8–5.2).^31^ The results were the same as those of Mtaya et al. in Tanzania (*n* = 387) (OR: 3.9, 95% CI: 2.9–5.2).^8^ These authors, using the Child-OIDP validated tools (Table 2), suggested that the oral problems were stronger influencers than other dimensions in the OHRQoL tool used, such as emotional and social well-being.^8,31^ When these studies were included in the meta-analysis, having oral problems (excluding dental caries) was still a factor likely to influence OHRQoL among children (Figure 3). Both the *I*^2^ test for heterogeneity favoured homogeneity of the studies (0.0%, *p* = 0.9) (Figure 4).

#### Socio-economic status

**FIGURE 5:**
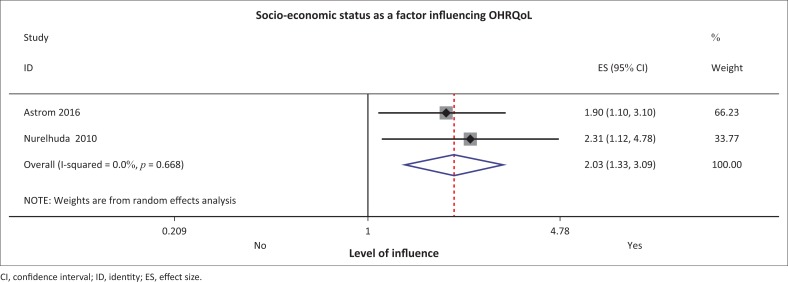
Forest plot for socio-economic status.

From the only longitudinal study in the review, with a sample of 2412 children in Tanzania, the authors concluded that an SES was 2.3 times more likely (95% CI: 1.12–4.78) to result in poorer OHRQoL (Figure 4), both used C-OIDP and OIDP tools (Table 2)^15^ Åstrøm et al. concluded that having parents who could afford dental care was a significant predictor of positive OHRQoL. Equally, in 2010 Nurelhuda et al. in their Sudanese study (*n* = 1109) found that SES, albeit in a cross-sectional study, was 1.9 times more likely (95% CI: 1.1–1.3) to influence OHRQoL.^15,30^ Meta-analysis of the two studies was displayed in the forest plot and supported the fact that a higher SES is 2.03 times more likely to influence OHRQoL outcomes (Figure 5).

#### Area of residence

**FIGURE 6:**
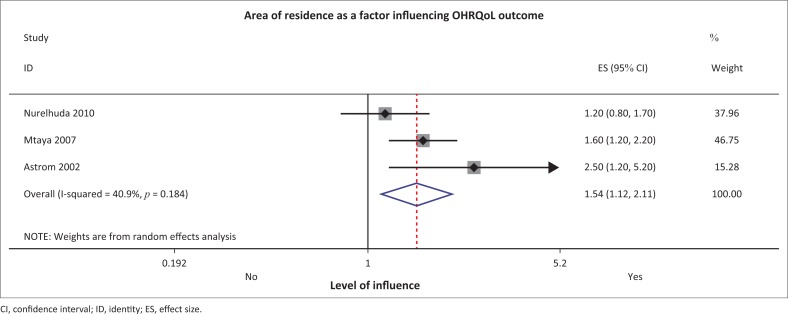
Forest plot ‘area of resident’ status influencing oral health-related quality of life.

The Tanzanian and Ugandan studies found the area of residence (district, rural and urban) to influence children’s oral HRQoL.^8,16^ Nurelhuda et al. reached a similar conclusion in the Sudanese study.^30^ The meta-analysis of these studies, with a combined sample of 3202 participants, supported the notion that the area or residence is a significant predictor of OHRQoL (Figure 6).

#### Satisfaction with oral health

**FIGURE 7:**
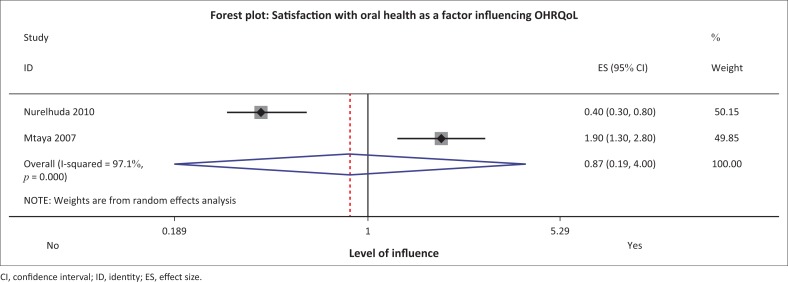
Forest plot for ‘satisfaction with oral health’ and oral health-related quality of life.

**FIGURE 8:**
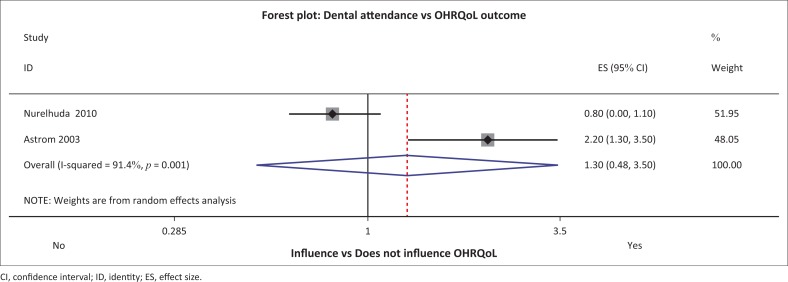
Forest plot for ‘dental attendance’ and oral health-related quality of life.

**FIGURE 9:**
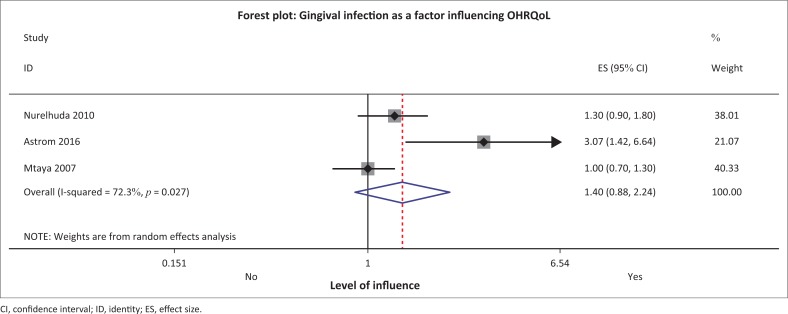
Forest plot for ‘gingival infection’ and oral health-related quality of life.

In the Sudanese and Tanzanian studies those children who were satisfied with their oral health status were less likely to report lower OHRQoL using the Child-OIDP tool (OR: 0.4, 95% CI: 0.3–0.6).^8,30^ However, the studies demonstrated moderate heterogeneity and the meta-analysis yielded contrasting findings. The overall effect was not significant with OR = 0.87 (95% CI: 0.19–4.00) with equal weighting of the studies (Figures 7–9).

#### Dental attendance and gingival infection

Both dental attendance and gingival infection were identified by Mtaya et al., Mashoto et al. and Nurelhuda et al. as factors influencing OHRQoL in their Tanzanian and Sudanese samples, respectively.^8,30,31^ The meta-analysis and pooling of results did not support their argument. Dental attendance had an overall effect of 1.30 (0.48–3.50), while gingival infection had an overall effect of 1.40 (0.88–2.24) (Figures 7–9).

### Seven more reported factors influencing oral health-related quality of life

Four studies, in addition to reporting factors similar to each other, also reported individual factors that were not cited by any other authors in this review. In the Sudan, among the visually challenged school attendants, it was found that *visual impairment* (*n* = 79, OR: 6.3, 95% CI: 1.7–22.7 [Results are significant]) significantly influenced OHRQoL.^29^ *Low self-efficacy* (*n* = 610, OR: 0.41, 95% CI: 0.19–0.89 [Results are significant; paper was included in meta-analyses]) and *depressed mental status n* = 428. OR: 3.23 (95% CI: 1.37–7.63 [Results are significant]) (paper was included in meta-analyses) were reported to be significant predictors of OHRQoL in the secondary schools learners in Tanzania.^15^ A Ugandan study by Åstrøm and Okullo cited that *religious affiliation* (*n* = 256, OR: 0.5, 95% CI: 0.3–0.9 [Results are significant; paper was included in meta-analyses]) and *missing teeth* (*n* =372, OR: 1.8, 95% CI: 1.0–3.2 [Results are significant; paper was included in meta-analyses]) significantly influenced the OHRQoL of adolescents using the OIDP tool, and were significantly influenced by OHRQoL instead.^15,16,30^

Of interest is that a study conducted among the Sudanese sample of 12-year-old school attendees showed a significant association between OHRQoL and behavioural factors, such as irregular tooth brushing and eating sugar sweetened snacks. ^[16,30]^ The findings included *tooth brushing frequency (irregular)* (OR: 1.0, 95% CI: 0.6–1.7), *plaque index* (*n* = 1045, OR: 1.3, 95% CI: 0.9–1.8) and *sugar sweetened snack intake* (*n* = 1045, OR: 1.4, 95% CI: 0.9–1.8), respectively.

## Discussion

This systematic review is the first aimed to critically analyse the factors influencing OHRQoL in African child populations. Oral health-related quality of life outcomes are usually assessed by measurement tools based on a conceptual framework which postulates that individual factors (demographic, personal biological and psychological status) and environmental factors (SES, dental access, caregivers status, education, type of residence and utilisation, and more) influence general health perceptions and the overall OHRQoL as postulated by Ferrens et al.^20^

All 10 articles included studies that used validated tools to measure OHRQoL in children (Table 2). The studies used good methods with regard to selection criteria of participants, validity and reliability of exposure and outcome variables as objectively assessed by the review involved (Y.M.-K., C.B.N., and T.R.D.R.). There was also a predisposition towards cross-sectional study designs in the studies selected. Cross-sectional studies cannot determine causality but only associations. However, these associations were rigorously determined especially when studies adjusted for confounders using regression analysis. Longitudinal studies including randomised controlled trials are obviously preferred as these designs have the potential to follow up on the exposure variable and can be used to determine causality.

The bias of studies towards East Africa could be attributed to one key author who contributed to eight of the 10 articles. The reason could be that the field on OHRQoL in dentistry is still at its development stage in Africa and the author is an expert author in the field.

*Dental caries*, the most common dental condition that affects 60% – 90% of school-aged children, in children more often results in pain and functional limitation.^31^ Thus, pain from severe dental decay can exert an impact on OHRQoL.^33^ The analysis in this review revealed an unexpected result regarding dental caries where it was not conclusively a factor that influenced OHRQoL. The result may be described by several explanations. Firstly, there was a high level of variation in the studies that reported on dental caries as a factor influencing OHRQoL. Heterogeneity in the studies within a systematic review is expected because a review combines studies that may be methodologically diverse; hence, it is more important to determine the extent to which heterogeneity affects the conclusions to the studies.^34^ A high percentage of *I*^2^ statistic (> 75%) indicates that there is an increased level of variability among the studies that could be because of the differences in their OHRQoL tools, studies adjusting for different confounders as well as the high number of studies included in meta-analysis which increases the percentage of the *I*^2^ statistic.^35^ Although all tools used were validated in the study settings, two tools (Child-OIDP and OIDP)^7,15,25,30,31,32^ were used where dental caries was reported to be a factor. Secondly, the heterogeneity could be attributed to the subjective nature of the design of OHRQoL tools that may be affected by cultural variation and differences in these East African countries. Thirdly, reporting of caries experience through DMFT/dmft without severity does not factor the element of pain. Indices such as Pulpal involvement, Ulcerations, Fistula and Abscess (PUFA) index do factor in the pain element, and thus may be useful in OHRQoL measurements.^36^ Fourthly, the DMFT/dmft index measures caries experience and is diluted by the filled and missing components related to treatment. It would be preferable for the analysis to single out the DMFT/dmft component to assess the active decay status when association analysis is performed. Lastly, the East African region is characterised by high fluoride content in the water source which makes teeth mottled and prone enough to initiation of dental caries, but the lesions are not severe because of the protective nature of fluorosis teeth.^37^

Dental caries was reported as a predictor of pain only if the prevalence and severity is high which tends to be common in the poorer communities who consume more refined carbohydrates diet as is common in South Africa.^23^ A Norwegian study by Koposovo et al. also supports the notion that the impact of dental caries on OHRQoL can be weak, and they attribute this finding to low general prevalence of caries in their Norwegian adolescent population.^38^

*Oral conditions other than dental caries* were a significant determinant of OHRQoL in this systematic review. These oral conditions maybe gingival or mucosal conditions, related to aesthetics or dental treatments. This is plausible as OHRQoL dimensions include pain, functional, psychological or emotional factors as well the social impacts. Malocclusion, which is an oral problem where there is misalignment or an incorrect relationship between the two dental arches, may result in aesthetic and functional problems. This is more likely to lead to a self-perceived treatment need which is common in malocclusion cases. It heightens the way the children perceive their own oral health. The children tend to have a worsened OHRQoL when they perceive that they ought to receive treatment, a perception that is shaped by the societal expectations. Appearance, which is affected in malocclusion cases, is very important in children’s lives particularly as they approach puberty. The notion is supported by Koposova et al.’s study in Europe among 12-year-olds where it was concluded that dental aesthetics was found to influence their OHRQoL.^38^

*Socio-economic status* measured by attending private versus public school was found to play an important role in influencing OHRQoL in the review. A higher SES may result in preventive visits and better access to dental health services and, thus, is likely to result in improved dental health, no pain, no early extraction and resulting malocclusion and a better OHRQoL. Thus, a better SES increases access to aesthetic services and may possibly influence the non-clinical dimensions (behavioural and social) of OHRQoL. Issues such as missing teeth, early extraction and later malocclusion are averted early, and thus improve oral health perceptions of adolescents. This finding in this review is congruent with literature where parents’ low-income is closely related to the availability of resources and children from low-income families are likely to have a lower OHRQoL.^39,40^ In addition, the study among 12-year-olds in Norway and Russia reported a poorer OHRQoL for the less privileged Russian children than their Norwegian counterparts.^38^ Furthermore, a study of 12-year-old Thai children of low socio-economic status found that they were likely to have a high level of dental caries with subsequent negative OHRQoL impact.^41^ So, this review in the African setting characterised by low-income countries with few resources also confirmed that resources will invariably influence OHRQoL. These findings highlight the context-related dimensions of most OHRQoL frameworks. Unfavourable social conditions and poor SES have a negative impact on children’s OHRQoL.^42^ Locker states that in the Canadian study, low SES scored worse than high SES.^43^

There was evidence linking *area of residence* with negative outcomes on OHRQoL in African child populations from this review. Mtaya et al. and Åstrøm and Okullo,^8,16^ in their Tanzanian and Ugandan studies, found the area of residence (rural vs. urban) to influence children’s OHRQoL.^6,16^ Nurelhuda et al. reached a similar conclusion in their Sudanese study.^30^ Rural districts tend to have poor availability and less access to dental facilities. Reduced access is likely to result in less preventive and curative services which might affect OHRQoL outcomes on various levels such as pain, functional and psychological impact. Children in urban dwellings tended to have or report better OHRQoL outcomes because of socio-demographic characteristics such as better parental education.^8^ One can argue that SES will be a mediating factor to the area (urban or rural) where children reside. If both factors have a strong relationship with the dependant variable, then there is a chain of risk factors that an even worse-off outcome regarding OHRQoL may prevail.^44^

*Satisfaction with oral health* is likely to result in a good self-rating on oral health and it is influenced by attitudes towards oral health. The result from this review showed moderate heterogeneity, and meta-analysis showed that this factor did not influence OHRQoL from the only two studies in the review. Satisfaction with oral health usually results from the individual’s attitudes; these attitudes are shaped by socialisation and context. In contrast, studies conducted outside Africa from literature have shown that oral health perceptions and attitudes of the caregiver or parents and of children themselves can influence the OHRQoL in children.^33,45^ Shaghaghian et al. reported that parental attitude to children’s oral hygiene habits influences children’s oral health status and their OHRQoL.^45^ Gomes et al. conclude that those caregivers who viewed their children’s oral health as poor were more likely to report a greater impact on OHRQoL.^33^ Different children in different geographical areas such as Saudi Arabia, Brazil and the United Kingdom responded differently especially on the social well-being and emotional well-being constructs of OHRQoL instruments, despite the translation and adaptation of the tool.^46,47,48,49^ The difference can be explained by embedded cultural influences. It is for this reason that the development of a conceptual equivalence of OHRQoL measurement tools is recommended before it can be used in settings different from those in which it originated.^9^

### More factors influencing oral health-related quality of life

*Physical handicap such as visual impairment* will impart an element of physical limitation, thus it may result in restrictive action and lack of visual–manual coordination. Despite the low sample in the reviewed study, of note among these visually impaired school attendees, those who were boarders had a poorer oral hygiene compared with non-boarders. Therefore, lack of assistance and supervision by caregivers may have mediated the resulting poorer oral hygiene. Less or no assistance with oral hygiene practices (e.g. toothbrushing and use of mouth guards) may thus lead to poor oral health states such as poor oral hygiene and traumatic dental injuries. A poor oral health status and the associated oral conditions may cause a negative reporting on OHRQoL.^29^ Irregular toothbrushing among the 12-year-old non-visually disabled Sudanese children in a similar setting did, however, not lead to a poor OHRQoL, perhaps the traumatic injures carried more weight in the OHRQoL reporting rather limited manual–visual brushing coordination.^30^

*Mental status is closely related to perception of self*; if individuals have less confidence and suffer from *depression* then their attitudes are generally negative.^50^ The reporting of OHRQoL is subjective in nature, thus the psychological dimension of the OIDP tool used among the Tanzanian school learners carried more weight than the clinical factors because of their depressive symptoms.^15^ The authors caution against a conclusion based on the mediating factor related to dental care utilisation, which was generally poor in the Tanzanian setting. However, a prerequisite for good oral health behaviour is self-efficacy to, for example, use floss, regular brushing and proper diet, according to the theory of planned behaviour.^51^ Self-efficacy may be lacking in the depressive states situations.

*Behavioural factors such as poor dietary habits and irregular brushing* on their own failed to make an impact on these African child populations; however, they may be mediated by the level of adolescents’ self-efficacy^15^ or by assistance or support in self-care.^29^ Socio-economic status is an important mediator or moderator in this instance because it will increase the dental attendance patterns and enhance preventive behaviour.

*Religion* such as reporting being Muslim was an important factor in the Ugandan adolescent sample as they were less likely to report oral impacts.^16^ Religion and spirituality are rarely reported in the health-related patient-reported outcomes. O’Connell and Skevington pointed out in their review that when the idea of spiritually is visited, it is usually as part of the social or psychological phenomenon of HRQoL outcomes and not a stand-alone dimension. Not enough is reported about religion, and it tends to be a salient concept in the OHRQoL.^52^

The issue of a *child’s age* as a determinant factor did not come up in the African studies in this systematic review, although Barbosa and Gaviao argue that the child’s age, development and gender influence and affect their well-being.^53^ Studies included in the review did adjust for age and gender, and in both instances there were no significant predictors. Except for two studies that used caregiver reports, most used adolescents groups of comparable ages (Table 2). Genderson et al. argues that the issue of self-concept is age-dependent and is heightened and important during adolescence because oral health is ‘strongly age-dependent’,^54^ hence there are differences between children and adults in OHRQoL measures.^55^

*Dental utilisation and access* did not influence OHRQoL in this review when results were pooled; this is in contrast to the Indian study by Kumar et al. which found that OHRQoL was better for participants who had been to the dentist within the past 12 months.^56^ Dental utilisation increases when dental access is enhanced. However, unlike in Kumar et al.’s^56^ Indian study, it was surprising in the Ugandan study that there was an inverse relationship where higher utilisation led to poorer OHRQoL reports.^16^ Nonetheless, following pooling the results in the meta-analysis, dental utilisation was not an influencing factor in this review. Introduction of free primary services in other African settings has shown the increased work operator load experience and thus poor services.^57^ In Uganda, the Lira District has a free user-fee policy for a public oral service in place. User-free policies may lead to poor services, thus affecting OHRQoL directly as the perceptions are rated by experience during dental visits.

A key finding in the articles reviewed was that factors influencing OHRQoL in children were environmental in nature (family SES and area of residence), but depended on the individual biological status and symptoms from oral problems. These oral problems, however, did not include dental caries, the most common oral condition. When assessing the findings against the Ferrans et al.^20^ and Sischo and Broder’s^13^ conceptual frameworks on OHRQoL, individual characteristics pertained only to the biological status and not the demographic factors. Sischo and Broder’s framework goes further to unpack the individual characteristics (Figure 1).^13,20^ There seems to be congruence with the Sischo and Broder explanation of the framework in this African setting where oral medical condition, psyche status (low self-efficacy and depressive states) and physical disability of the children were the significant individual factors to influence. However, the environmental issues arising from the review were the area of residence and SES only. The parent or caregiver status was implied by the family’s SES status.

The factors in the review were balanced between the environmental and individuals’ oral problems, but the pathway did not necessarily fit the models (Figure 1). Overall perceptions with oral health were not evident. Other factors such as oral health behaviour, perception or satisfaction with oral health and dental access were significant in other settings in high-income countries but not evident in the African setting. This may be related to contextual importance of factors or perhaps these factors were reported by few studies.^58^ It is evident from the review that the OHRQoL measurement tools do not tease out in the setting what is reported in high-income country settings. The form and degree of impacts could vary between populations with different cultural backgrounds.^59^ The question is: How are issues framed and from whose point of view that they fail to capture or be relevant in the setting?

Moreover, these systematic review findings are in contrast with the Locker model of OHRQoL, which suggests that mainly physical symptoms and functional limitation dimensions will carry weight rather than environmental dimensions in influencing OHRQoL.^19^ The biomedical Locker model hypothesised that oral disease will result in ‘pain’, ‘impairment’ and functional limitation. These constructs will, in turn, lead to physical and psychological disability, handicap, and thus affect the overall OHRQoL.^19^ Thus, in this African setting context the contributing factors spanned the socio-environmental context in nature and biomedical dimensions.

Oral health-related quality of life does not exist in a vacuum, but is influenced by interplay of socio-economical, biological and personal psychosocial factors. Herdman et al. argue convincingly that cross-cultural adaptation is warranted as it guards against automatic assumptions that OHRQoL domains important to one culture will be equally relevant in all cultures.^9^ Comprehension of this interplay will begin a process to assess the OHRQoL impacts, hence the importance of studying the factors of OHRQoL. Understanding influencing factors will assist in planning so that measures to reduce OHRQoL impacts are incorporated in the integrated management of children’s oral health in Africa.

### Limitations

Mostly cross-sectional studies were retrieved from this review given that research into OHRQoL is fairly recent in Africa. However, data analysis included accounting for possible confounders when looking for associated factors related to OHRQoL in children. The reporting of ORs implied that authors dichotomised the OHRQoL outcome describing the absence or presence of a negative impact. Reporting the presence of OHRQoL impacts in binary form makes it impossible to see the intensity of the OHRQoL impacts.

## Conclusion

There is preliminary evidence to suggest that in Africa there is an association between individual factors such as children’s psyche and oral problems, excluding dental caries, and contextual social determinants such as area of residence and SES and children’s OHRQoL in African contexts. Thus, in this African setting context the contributing factors spanned the socio-environmental context in nature and biomedical dimensions. There seemed to be a contextual viewpoint underpinning the current OHRQoL frameworks and OHRQoL was context-reliant.

There is evidence of rigorous work in the field of OHRQoL in Africa. However, most literature is dominated by quantitative prevalence studies. More work in qualitative and longitudinal studies can assess causality in this field in the setting to see if factors related to OHRQoL are context-reliant.
